# Long COVID Mechanisms, Microvascular Effects, and Evaluation Based on Incidence

**DOI:** 10.3390/life15060887

**Published:** 2025-05-30

**Authors:** Aristotle G. Koutsiaris, Kostas Karakousis

**Affiliations:** 1 Faculty of Medicine, School of Health Sciences, University of Thessaly, Biopolis Campus, 41500 Larissa, Greece; 2 Department of Internal Medicine, General Hospital of Larissa, 41221 Larissa, Greece; karakoussis@yahoo.gr

**Keywords:** COVID, long, microcirculation, mechanism, microvascular loss, pathophysiology, blood supply, incidence

## Abstract

Since the initial reports of Long COVID symptoms, numerous pathophysiological mechanisms have been proposed to explain them; nevertheless, no consensus has been reached. Some of these mechanisms are directly linked to microcirculation, while others are related indirectly. Those with a direct connection involve the respiratory system (such as pulmonary embolism), the cardiovascular system (including cardiac arrest, heart failure, myocardial inflammation, stroke, endothelial dysfunction, and microangiopathy), hematological conditions (like coagulopathy, deep vein thrombosis, microclots, and endothelial irregularities), and brain function. However, few of these mechanisms are grounded in quantitative data and fundamental physiological principles. Furthermore, diagnostic and therapeutic methods remain inadequate. This report provides a brief overview of these processes, focusing primarily on quantitative data, recently proposed mechanisms, and advances in microcirculation, with a special emphasis on the tissue blood supply reduction (TBSR or SR in short) mechanism. Then, the SR pathophysiological mechanism is assessed based on the total incidence rate of the Long COVID symptoms that can be directly attributed to this mechanism. The proposed SR mechanism can account for seven principal Long COVID symptoms with a total normalized incidence of 76%.

## 1. Introduction

Even if the world is not in an emergency, the confirmed cases and death count from COVID-19 still rise. Over 777 million confirmed cases and over 7 million deaths have been reported globally, and wastewater surveillance shows that circulation is approximately 2 to 19 times higher than reported cases [1].

After 5 years from the start of the COVID-19 pandemic, the exact pathophysiological mechanism of the disease is still unknown even though it is now recognized that it is a triphasic disease with three overlapping stages of **(a)** viral replication in the upper respiratory tract with direct invasion into the alveolar epithelium and common symptoms of fever, fatigue, cough, and sore throat, **(b)** hyperinflammatory cytokine storm with low oxygen levels, shortness of breath, and acute lung damage (COVID-19 pneumonia), and **(c)** hypercoagulability or thrombosis which can lead to severe respiratory failure and multi-organ damage [2].

About 5% of infected, not previously vaccinated individuals become critically ill [3] and are admitted to the intensive care unit, where they can be treated by a variety of non-pharmacological respiratory support strategies, among which is mechanical ventilation. In a prospective one-year-long observational study [4] including 91 critically ill COVID-19 patients, the C-reactive protein and fibrinogen were shown as independent predictors of mortality.

Regarding the last two stages of the acute phase, a significant connection to the microcirculation exists [5,6,7]. In addition, loss of red blood cell deformability and systemic endothelialitis have been associated with COVID-19 microthrombosis [8]. Endothelialitis as a consequence of the endothelial virus invasion can be the cause of the disseminated microvascular dysfunction, microthrombosis, ischemia, tissue hypoxia, and oedema, resulting in multi-organ failure [7,9,10]. It has been reported that COVID-19 coagulopathy is different from disseminated intravascular coagulation during bacterial infections and sepsis [11].

About 10% of the infected individuals, after the acute phase (with one or more of the three stages depending on the severity) of the COVID-19 disease, develop a condition of persistent symptoms known as Long COVID [12] with a prolonged recovery phase that can take weeks and up to many months. The order of incidence for nine Long COVID symptoms, in a 6-month period after COVID-19 diagnosis, is anxiety/depression (23%), abnormal breathing (19%), abdominal symptoms (16%), fatigue/malaise (13%), chest/throat pain (13%), other pain (12%), headache (9%), cognitive symptoms (8%), and myalgia (3%) [13]. It is noted that all nine symptoms occur more frequently after COVID-19 than after influenza (*p* < 0.001) and symptom incidence rates come from a study on 273,618 COVID-19 survivors [13].

Furthermore, the risk of developing Long COVID symptoms is higher in patients with more severe COVID-19 [13]. In a recent magnetic resonance imaging study [14], a lower microvascular perfusion was found in intensive care unit COVID-19 survivors compared to general ward patients. In an ophthalmic imaging study [15], the severity of retinal microvascular alterations was positively associated with cognitive impairments in post-COVID individuals.

In this narrative review, after the introduction (Section 1), a summary of the currently available data linking Long COVID and microcirculation is given (Section 2). Then, a brief overview of the numerous proposed Long COVID pathophysiological mechanisms is made (Section 3) with a special emphasis on microvascular effects, microthrombosis, and the related tissue blood supply reduction (TBSR or SR in short) mechanism. Then, the SR pathophysiological mechanism is assessed (Section 4) based on the total incidence rate of the Long COVID symptoms that can be directly attributed to this mechanism. Finally, there is a short conclusion section (Section 5).

This paper aims to give a brief presentation of the proposed Long COVID mechanisms, emphasizing close associations with microcirculatory disorders and related quantitative data from case-control studies. An additional goal is to describe the basic structure of the SR mechanism and introduce a logical context for quantifying an incidence-based connection between the SR mechanism and the principal symptoms of Long COVID. Along the way, a few suggestions are made, and a new term is proposed for endothelial cells.

## 2. Long COVID and Microcirculation

**Figure 1:**
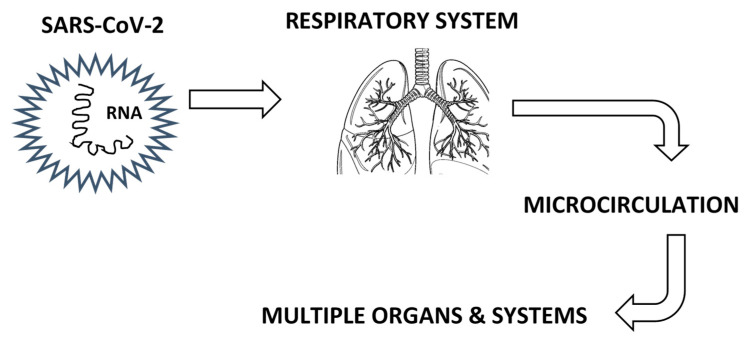
Vascular effects after SARS-CoV-2 infection.

Microcirculation can be affected by many conditions “out of the normal state”, such as pregnancy [16]. COVID-19 was reported to affect the microcirculation of many different organs [17], including the lung, kidney, brain, eye, skin, and the heart (Figure 1).

However, direct in vivo observation of the human microcirculation is limited to a few tissues such as the eye, nailfold, and the sublingual tissue. Even though a considerable advancement in human microvascular hemodynamic measurements was made in the last decades [18], it is still difficult to take such measurements accurately and reliably [19]. The same was true for measuring microvascular density, until the advent of optical tomography angiography (OCTA), which is now considered “standard” technology for an ophthalmology department [20]. The automatic quantification of vascular density gives a valuable tool to the clinical doctors who want to see how the microvasculature is affected by various physiological [21,22] and clinical conditions [23,24,25,26,27].

OCTA is the main contributing factor to the high availability of microvascular loss (ML) data in post-COVID patients [15,28,29], and numerous researchers have reported significant case-control microvascular loss [30,31,32,33,34,35,36,37,38]. It is noted that in a recent meta-analysis of retinal microvascular changes in post-COVID patients, including all available case-control OCTA studies both with and without significant results [39], it was shown that, overall, there was a statistically significant microvessel density reduction and foveal avascular zone enlargement, or, in short, a significant microvascular loss (ML). Retinal vessel diameter narrowing as a result of Long COVID chronic inflammation was also reported in a recent narrative review [40], and the importance of studying viral infections on retinal microcirculation was emphasized since microvascular alterations appear earlier than changes in larger vessels. In addition, a significant hemodynamic reduction [41] was reported in the eyes of post-COVID patients.

Furthermore, many case-control studies reported significant microvascular loss and hemodynamic reduction in the skin [42,43,44,45] and sublingual tissue [46] of post-COVID patients.

## 3. Proposed Long COVID Pathophysiological Mechanisms

### 3.1. Respiratory System

Regarding the respiratory system, abnormal breathing and chest/throat pain are the second (19%) and fifth (13%) most commonly reported Long COVID symptoms, respectively [13] (Table 1 and Figure 1). The coronavirus infection, with mild to severe symptoms, dysregulates vascular permeability in the lungs and causes a ‘cytokine storm’ along with profound liquid accumulation in the lungs, which turns breathing into an agonizing task [3]. Ιn a case-control study with 100 post-COVID patients, small airways disease (ground-glass opacities and air trapping) was quantified by image processing on chest CT scans and occurred independently of initial infection severity [47]. Littlefield et al. [48] found that pulmonary Long COVID is associated with increased levels of SARS-CoV-2-specific T cells, systemic inflammation, and reduced lung function. The respiratory Long COVID following post-COVID pneumonia is characterized by microvascular injury identified by the reduction of lung capillary blood volume [49]. All aforementioned reports are associated with a respiratory pathophysiological mechanism that is directly related to the microvasculature malfunction (Table 1).

### 3.2. Immune System

Regarding the immune system, it is clearly implicated in the acute phase of the disease, including viral replication and hyperinflammatory response. Moreover, many Long COVID studies have reported immune dysregulation regarding T cells, CD4+ and CD8+ effector memory cells, naïve T and B cells, expression of type I and type III interferons, IL-4- and IL-6-secreting CD4+ T cells, dendritic cells, levels of cytokines (IL-1β, IL-6, TNF and IP10), C-reactive protein (CRP), levels of IgG and IgM, of spike-specific memory B cells, of nucleocapsid IgG, and of spike-specific IgG [12,50]. This is an indication of a persisting inflammatory process after the acute phase of the COVID-19 disease.

Other studies suggested autoimmune reaction as the underlying cause of Long COVID, under the premise that SARS-CoV-2 shares structural similarities with human proteins (molecular mimicry).

### 3.3. Viral Persistence

Viral persistence (RNA or protein viral remnants in various tissues) was detected in postmortem tissues [51] and suggested as a mechanism of Long COVID symptoms [12,50,52]. This mechanism can be related to many physiological systems of the body. Viral persistence and reactivation (such as those of herpesviruses including Epstein–Barr Virus, human herpesvirus 6, and others) have been associated with myalgic encephalomyelitis/chronic fatigue syndrome (ME/CFS) and various malignancies. Fatigue/malaise and myalgia are the fourth (13%) and ninth (3%) most commonly reported Long COVID symptoms, respectively [13].

### 3.4. Nervous System

Anxiety/depression, other pain (unspecified), headache, and cognitive symptoms are the first (23%), sixth (12%), seventh (9%), and eighth (8%) most commonly reported Long COVID symptoms, respectively [13]. SARS-CoV-2 can affect the nervous system through the olfactory, trigeminal, and vagus nerves or the blood–brain barrier by disrupting the neurovascular unit [53]. Several neurological mechanisms have been proposed as the underlying cause of Long COVID.

First, the disruption of the blood–brain barrier [53,54] allows inflammatory molecules to enter the brain, leading to neuroinflammation, cognitive dysfunction, and symptoms such as “brain fog”. In addition, disruption of the choroid plexus barrier and microvascular damage have been reported in mice [55]. This mechanism is directly linked to the microvascular system.

Second, oxidation-driven biochemical remodeling of ryanodine receptor isoform 2 (RyR2) channels, inducing intracellular Ca^2+^ leak, was proposed as a mechanism [56]. The ryanodine receptor family is one of the two families of calcium release channels in the endoplasmic reticulum membrane. According to this proposition, SARS-CoV-2 infection oxidizes the RyR2 channel, causing calstabin2 depletion, destabilization of the channel’s closed state, and endoplasmic reticulum calcium leak contributing to cardiac dysfunction, pulmonary insufficiency, and cognitive abnormalities associated with Long COVID and neurodegeneration (Alzheimer’s disease). However, in their work [56] the RyR2 channel was immunoprecipitated from brain, lung, and heart tissue of only 10 patients who have succumbed to COVID-19.

Third, cytotoxic amyloid aggregates (assemblies) of SARS-CoV-2 proteins or peptides were proposed as a mechanism triggering neurological symptoms in COVID-19 [57]. This is also a mechanism related to neurodegenerative diseases such as Alzheimer’s and Parkinson’s.

Fourth, SARS-CoV-2 spike (S1) and nucleocapsid (N) protein levels in plasma neuron-derived extracellular vesicles (EVs) and astrocyte-derived EVs were higher in all post-COVID subgroups than in the controls [58]. In addition, abnormal levels of mitochondrial proteins in plasma neuron-derived and astrocyte-derived EVs in all post-COVID subgroups were correlated with post-COVID neuropsychiatric manifestations and were proposed as biomarkers for long-COVID prognostics and therapeutic trials [58]. Furthermore, loss of mitochondrial membrane potential was reported in Long COVID patients [59]. SARS-CoV-2 can infect and replicate in brain organoids [60], and small-fiber neuropathy was observed in patients with Long COVID [61].

All the above proposed mechanisms for Long COVID can be linked to the autonomic nervous system dysfunction (dysautonomia) [62] which is commonly found in Long COVID together with postural orthostatic tachycardia syndrome (POTS) [63,64]. Disrupted autonomic control of circulation is the cause of cardiovascular autonomic dysfunction (CVAD), which is most commonly presented by POTS and inappropriate sinus tachycardia. Dysautonomia and CVAD as Long COVID components are a major health-care burden [65].

### 3.5. Gastrointestinal System

Abdominal problems are the third (16%) most commonly reported Long COVID symptoms [13]. Dysbacteriosis in the gut persists after clearance of the COVID-19 virus, and COVID-19 patients are depleted in gut bacteria with immunomodulatory potential [66]. Compositional gut microbiome alterations in Long COVID patients with low levels of butyrate-producing bacteria were reported 6 months after infection [67]. In addition, persistent respiratory symptoms and neuropsychiatric symptoms correlated with gut pathogens [67].

It has been proposed that gut dysbiosis (e.g., from antibiotic use throughout life) may be a risk factor for ME/CFS, with other possible pathophysiological risk factors of altered gut–brain axis activity, increased gut permeability, reduced levels of short-chain fatty acids, D-lactic acidosis, abnormal tryptophan metabolism, and low activity of the kynurenine pathway [68]. It was suspected that an altered microbiome through the gut–brain axis may contribute to neurocognitive impairments of ME/CFS patients [68]. Similarly, for the Long COVID case, gut dysbiosis due to viral persistence (see Section 3.3) may contribute to the development of ME/CFS.

### 3.6. Cardiovascular System and Microcirculation

#### 3.6.1. In Vitro Studies and Blood Coagulation

As with COVID-19, Long COVID has been associated with endothelial damage, deep vein thrombosis, bleeding, red blood cell damage, and microclots both in vivo [37] and in vitro [69]. In a recent case-control in vitro study [70], they found significantly elevated plasma levels of von Willebrand factor (VWF) and Factor VIII in gynecologic Long COVID patients. They also found persistently elevated VWF and Factor VIII concentrations for at least 2 years, which correlates well with other reports of thrombotic microangiopathy, taking into account the importance of these factors in the coagulation pathway. In a retrospective analysis of laboratory data from 1,429 COVID-19 hospitalized patients [71], impressively elevated values of D-dimer, procalcitonin, and C-reactive protein (CRP) were reported. However, it would be very exciting to see the same data analysis only on the 103 patients in Table 17 of ref. [71] with normal procalcitonin levels, excluding in this way the confounding factor of bacterial infection.

#### 3.6.2. The Endothelium

Baldassarro et al. [72] demonstrated in vitro, in endothelial capillary cells derived from different body organs, that SARS-CoV-2 peptides induce endothelial-to-mesenchymal transition, corroborating the premise of the COVID-19-mediated endothelial microvascular damage. Gultom et al. [73] showed in vitro that SARS-CoV-2 spike protein generated prolonged inflammatory responses in both the macro (aortic) and microvascular (pulmonary) endothelial cells. In vitro micro-models [74] can provide valuable information on how the virus is transmitted through the air–blood interface of the lung alveolus as long as they mimic adequately the physiological conditions.

Dysregulation of the endothelial barrier in the acute phase causes endothelialitis, hypercoagulation, and hypofibrinolysis which may persist for a long time manifesting as Long COVID [75,76]. Stahlberg et al. [77] reported peripheral microvascular endothelial dysfunction associated with NT-ProBNP levels in post-COVID patients more than 6 months after the acute infection.

The exact way in which the endothelial barrier is dysregulated is difficult to delineate because of the complexity of the endothelial cell [78]. Except for specific biomolecules, biomechanical signals, such as wall shear stress [79,80], cyclic strain, and hydrostatic forces [81], act as inputs to the endothelial cell which can express a variety of outputs including proliferation, cell shape modification, and the release of a gamut of substances affecting vasomotor tone, hemostatic balance, leukocyte trafficking, and others. In other words, endothelial cells do not act only as sensors and processors but also as effectors with output signals producing specific effects, and therefore they act as “cardiovascular sensors, processors, and effectors” (CSPEs).

Moreover, the endothelium is not simply a barrier but is already considered an endocrine organ [82]. The situation is further complicated by the fact that there is a considerable segmental heterogeneity; that is, an endothelial phenotypic and functional difference among different segments of the vascular system [79,83].

#### 3.6.3. Microcirculation

With a focus on microcirculation, Cutolo et al. [84] proposed two non-specific nailfold capillaroscopic patterns of COVID-19 microvascular abnormalities: an “early” pattern appearing in the acute phase characterized by abnormal microvessel shapes, microhaemorrhages, and microthromboses, followed by a “late” pattern, in COVID-19 survivors, mainly characterized by loss of capillaries.

However, the aforementioned proposed Long COVID mechanisms, from all physiological systems, did not include quantifiable metrics in a pathophysiological context based on fundamental physical principles.

**Figure 2:**
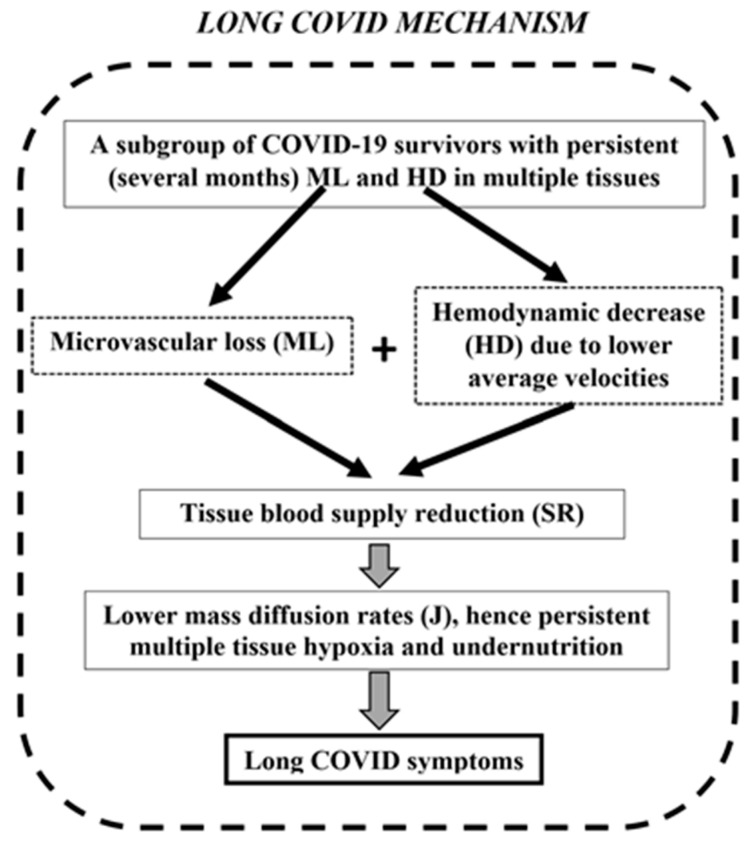
The proposed Long COVID pathophysiological mechanism (image modified from Koutsiaris [85]). The common framework combines microvascular loss (ML) and hemodynamical decrease (HD) to a single index called tissue blood supply reduction (TBSR or SR in short).

Recently, a pathophysiological mechanism for Long COVID was proposed [85], based on tissue blood supply reduction (TBSR or SR in short), which was estimated mathematically from microvascular measurements linked to the physiological principle of the velocity-diffusion equation [86]. The main idea for the proposition of this mechanism was that when a hemodynamic decrease (HD) is reported, this decrease refers to the remaining microvessels of the measurement site. However, this is only a fraction of the total blood supply loss of the local tissue since there is also a microvascular loss (ML) which should be taken into account in combination with the HD (Figure 2).

For the ML quantification of case-control studies, the following metrics were introduced [85]: vessel density reduction (VDR), foveal avascular zone enlargement (FAZE), capillary density reduction (CDR), and perfused vessel reduction (PPVR). These ML metrics were combined mathematically with HD in a single index called tissue blood supply reduction (SR) that better describes the total microvascular blood supply effect of every disease on body tissues (Figure 2). After gathering ML and HD case-control data from different geographical areas and multiple tissues, an SR estimation of 47% was reported from a total of 634 post-COVID patients (Table 2).

A significant SR has as a consequence much lower diffusion rates (Figure 2) and therefore multiple tissue hypoxia and undernutrition that can cause most Long COVID symptoms, among which many are related to brain function, such as cognitive impairment and headache. This fits very well with a recent case-control study [87] which reported elevated cerebral oxygen extraction fraction (OEF) in patients with post-COVID neurological conditions because a high OEF is a feature of microvascular flow insufficiency affecting oxygen homeostasis. Since three core Long COVID symptoms (anxiety/depression, headache, cognitive impairment) are directly related to the brain, there is also a direct connection to the SR mechanism due to the known sensitivity of the brain to oxygen supply (Table 3).

Diminished intracellular oxygen delivery is supported additionally by studies on red blood cells [8,88]. In a recent case-control study [88], Long COVID patients demonstrated decreased peripheral tissue oxygenation and a correlation of adenosine triphosphate (ATP) concentration in their erythrocytes with markers of systemic inflammation reactivation.

Furthermore, diminished intracellular oxygen delivery is supported at a whole-body level by a series of studies on exercise performance [89,90,91]. Lafeta et al. [90] reported exercise intolerance in a study with 87 COVID-19 survivors after hospitalization, and a hypothesis of lung microvascular injury was made. Associations of Long COVID to reduced measures of exercise performance were also reported recently [89,91], which supports the pathophysiological mechanism of tissue blood supply reduction (SR).

Reduced exercise performance is also directly related to chronic fatigue syndrome. Myalgic encephalomyelitis/chronic fatigue syndrome (ME/CFS) has been associated with Long COVID [92], and it has been hypothesized that the interaction of precapillary cardiovascular disturbances with primary microcirculatory capillary disturbances causes Long COVID and the associated ME/CFS [93]. This hypothesis, together with the SR mechanism, gives a direct connection of ME/CFS to microcirculation. Fatigue/malaise and myalgia are the fourth (13%) and ninth (3%) most commonly reported Long COVID symptoms, respectively [13] (Table 4).

#### 3.6.4. Heart and Large Vessels

Some studies implicate the heart and large vessels in the pathophysiology of Long COVID. Panagiotides et al. [94] have hypothesized on myocardial stiffness, edema, and impaired contractility due to endothelial damage and glycocalyx disintegration in COVID-19 and Long COVID. In a recent case-control study [95], an increased arterial stiffness and carotid-radial pulse wave velocity were reported in individuals with Long COVID 1–9 months after infection. In another case-control study [96], an impaired left ventricular function was reported in COVID-19 patients after hospitalization, assessed by exercise stress echocardiography. A recent mathematical study integrating the cardiovascular and immune systems [97] found a significant reduction in the left ventricular ejection fraction (LVEF) in the case of COVID-19 survivors, and a LVEF reduction well below the normal levels in the case of non-survivors. These results correlate well with the reduced exercise performance of post-COVID patients and the SR mechanism.

## 4. SR Mechanism Evaluation Based on Long COVID Symptom Incidence

Table 5 shows the incidence found by Taquet et al. [13] for nine core Long COVID symptoms in decreasing order. In some patients, more than one symptom occurred (co-occurrence), so the sum of the reported incidences was more than 100%. In this work, the reported incidences were normalized for comparative purposes. The sum of the normalized incidences for the nine core Long COVID symptoms is 100%, as shown in the third column of Table 5.

An evaluation of the SR mechanism could be based on the cumulative normalized incidence rate of the Long COVID symptoms that can be directly attributed to this mechanism. As shown in the previous sections, seven long-term COVID symptoms can be directly related to the tissue blood supply reduction (SR) mechanism through the respiratory (Table 1), nervous (Table 3), and nervous/muscular/microvascular (Table 4) systems. Each Long COVID symptom, with its corresponding physiological system and normalized incidence, is shown in Table 6. In this logical context, the proposed SR mechanism can account for 7 of the 9 principal Long COVID symptoms, with a total normalized incidence of 76%.

A limitation of this evaluation analysis is a residual of 24% of the normalized incidence rate that is not directly associated with this mechanism. This 24% corresponds to the sum of 14% (from “abdominal symptoms” (Table 5)) and 10% (from “other pain” (Table 5)). Regarding the 14% from abdominal symptoms, however, a potential link to microthrombosis may exist through the degradation of the intestinal epithelial cell junctional proteins and the biochemical barrier to the microvessels [85]. In addition, gut dysbiosis due to viral persistence may contribute to the development of ME/CFS (see Section 3.5). Regarding the 10%, however, unspecified pain could be caused by systemic microthrombosis or by tissue blood supply reduction in the brain’s pain center.

## 5. Conclusions

Since the initial reports of Long COVID symptoms, different pathophysiological mechanisms have been suggested to explain them, but no consensus has been reached. Furthermore, diagnostic and therapeutic methods remain inadequate. This work provides a brief overview of these mechanisms, with a primary focus on quantitative data and recent advances in microcirculation. A special emphasis is given on the tissue blood supply reduction (SR) mechanism [85], which is based on quantitative data from post-COVID patients (Table 2) in the context of fundamental physiological principles.

On the surplus, an assessment of the SR pathophysiological mechanism is given based on the incidence rates of the reported Long COVID symptoms that can be directly attributed to this mechanism. It seems that in the search for the pathophysiological mechanism behind Long COVID, we are close to a proposal that explains the vast majority of the reported symptoms since the SR mechanism can be linked directly to seven principal Long COVID symptoms with a total normalized incidence of 76% (Table 6).

## Figures and Tables

**Table 1:** Incidence of the respiratory Long COVID symptoms directly related to the microcirculation (incidence values from Taquet et al. [13]).

Physiological System	Long COVID Symptom	Incidence (%)
Respiratory	Abnormal breathing	19
	Chest/throat pain	13

**Table 2:** Tissue blood supply reduction (SR) from 634 post-COVID patients [85].

Metric	Average (%)	Tissue	N
HD	37	Conjunctiva/Skin/Brain	72
ML	16	Retina/Choroid/Sublingual	562
**SR**	**47**	**Multiple Tissues**	**634**

Data refer to a post-COVID period of 0 to 6 months. N: number of post-COVID patients, HD: hemodynamic decrease, ML: microvascular loss, SR: tissue blood supply reduction.

**Table 3:** Incidence of the cerebral Long COVID symptoms directly related to the microcirculation (incidence values from Taquet et al. [13]).

Physiological System	Long COVID Symptom	Incidence (%)
Nervous	Anxiety/Depression	23
	Headache	9
	Cognitive	8

**Table 4:** Incidence of the ME/CFS Long COVID symptoms directly related to microcirculation (incidence values from Taquet et al. [13]).

Physiological System	Long COVID Symptom	Incidence (%)
Nervous/Muscular/ Microvascular	Fatigue/Malaise (ME/CFS)	13
	Myalgia (ME/CFS)	3

ME/CFS: Myalgic encephalomyelitis/chronic fatigue syndrome.

**Table 5:** Normalized incidence for 9 core Long COVID symptoms (incidence values in the middle column from Taquet et al. [13]).

Long COVID Symptom	Incidence (%)	Normalized Incidence (%)
Anxiety/Depression	23	20
Abnormal breathing	19	16
Abdominal symptoms	16	14
Fatigue/Malaise	13	11
Chest/Throat pain	13	11
Other pain	12	10
Headache	9	8
Cognitive	8	7
Myalgia	3	3
**TOTAL**	**116**	**100**

**Table 6:** Normalized incidence of seven Long COVID symptoms directly related to the tissue blood supply reduction (SR) mechanism.

Physiological System	Long COVID Symptom	Normalized Incidence (%)
Respiratory (Table 1)	Abnormal breathing	16
	Chest/Throat pain	11
Nervous (Table 3)	Anxiety/Depression	20
	Headache	8
	Cognitive	7
Nervous/Muscular/ Microvascular (Table 4)	Fatigue/Malaise (ME/CFS)	11
	Myalgia (ME/CFS)	3
**TOTAL**		**76**

ME/CFS: Myalgic encephalomyelitis/chronic fatigue syndrome.

## Data Availability

No new data were created in this study. The original contributions presented in the study are included in the article.

## References

[1] WHO (2025). COVID-19 Epidemiological Update, Edition 175.

[2] Aldous C., Gkioulekas E., Oldfield P., Varon J., Marik P.E., Rendell M., Iglesias J., de Souza C., Prabhudesai P. (2024). Controversies in the Pandemic.

[3] Machado N.R., Dias K.T., Cortes B.F.S., Rodrigues S.F. (2023). Effect of Coronaviruses on Blood Vessel Permeability: Potential Therapeutic Targets. Ther. Adv. Respir. Dis..

[4] Zlojutro B., Jandric M., Momcicevic D., Dragic S., Kovacevic T., Djajic V., Stojiljkovic M.P., Skrbic R., Djuric D.M., Kovacevic P. (2023). Dynamic Changes in Coagulation, Hematological and Biochemical Parameters as Predictors of Mortality in Critically Ill COVID–19 Patients: A Prospective Observational Study. Clin. Hemorheol. Microcirc..

[5] Karahan S., Aydin K., Cetinkaya A., Sirakaya H.A. (2022). Nailfold Videocapillaroscopy in Patients with COVID-19-Associated Pneumonia in Intensive Care Units. J. Coll. Physicians Surg. Pak..

[6] Malgaj Vrečko M., Aleš-Rigler A., Borštnar Š., Večerić-Haler Ž. (2024). Coronavirus Disease 2019-Associated Thrombotic Microangiopathy: A Single-Center Experience. Int. J. Mol. Sci..

[7] Rosei C.A., Gaggero A., Famà F., Malerba P., Chiarini G., Nardin M., Brami V., Rossini C., Coschignano M.A., Porteri E. (2022). Skin Capillary Alterations in Patients with Acute SarsCoV2 Infection. J. Hypertens..

[8] Jung F., Connes P. (2024). Morphology and Function of Red Blood Cells in COVID-19 Patients: Current Overview 2023. Life.

[9] Kelliher S., Weiss L., Cullivan S., O’Rourke E., Murphy C.A., Toolan S., Lennon Á., Szklanna P.B., Comer S.P., Macleod H. (2022). Non-severe COVID-19 Is Associated with Endothelial Damage and Hypercoagulability Despite Pharmacological Thromboprophylaxis. J. Thromb. Haemost..

[10] Østergaard L. (2021). SARS CoV-2 Related Microvascular Damage and Symptoms during and after COVID-19: Consequences of Capillary Transit-time Changes, Tissue Hypoxia and Inflammation. Physiol. Rep..

[11] Russu E., Arbănaşi E.-M., Șchiopu A. (2024). Special Issue “COVID-19 Coagulopathy: Advances on Pathophysiology and Therapies”. Int. J. Mol. Sci..

[12] Davis H.E., McCorkell L., Vogel J.M., Topol E.J. (2023). Long COVID: Major Findings, Mechanisms and Recommendations. Nat. Rev. Microbiol..

[13] Taquet M., Dercon Q., Luciano S., Geddes J.R., Husain M., Harrison P.J. (2021). Incidence, Co-Occurrence, and Evolution of Long-COVID Features: A 6-Month Retrospective Cohort Study of 273,618 Survivors of COVID-19. PLoS Med..

[14] Van Der Knaap N., Klinkhammer S., Postma A.A., Visser-Meily J.M.A., Horn J., Van Heugten C.M., Voorter P.H.M., Van Der Thiel M.M., Drenthen G.S., Backes W.H. (2025). Post-COVID Microvascular Dysfunction in Hospitalized COVID-19 Survivors Is Associated with Acute Disease Severity and Persistent Cognitive Complaints. J. Neurol. Sci..

[15] Ariza M., Delas B., Rodriguez B., De Frutos B., Cano N., Segura B., Barrué C., Bejar J., Asaad M., Cortés C.U. (2024). Retinal Microvasculature Changes Linked to Executive Function Impairment after COVID-19. J. Clin. Med..

[16] Moka S., Koutsiaris A.G., Garas A., Messinis I., Tachmitzi S.V., Giannoukas A., Tsironi E.E. (2020). Blood Flow Velocity Comparison in the Eye Capillaries and Postcapillary Venules between Normal Pregnant and Non-Pregnant Women. Microvasc. Res..

[17] Karakasis P., Nasoufidou A., Sagris M., Fragakis N., Tsioufis K. (2024). Vascular Alterations Following COVID-19 Infection: A Comprehensive Literature Review. Life.

[18] Koutsiaris A.G. (2016). Hemodynamics in the Microcirculation. Ann. Biomed. Eng..

[19] Koutsiaris A.G. (2022). Meta-Analysis of Conjunctival Microvascular Hemorheology Metrics. Microvasc. Res..

[20] Koutsiaris A.G., Batis V., Liakopoulou G., Tachmitzi S.V., Detorakis E.T., Tsironi E.E. (2023). Optical Coherence Tomography Angiography (OCTA) of the Eye: A Review on Basic Principles, Advantages, Disadvantages and Device Specifications. Clin. Hemorheol. Microcirc..

[21] Fang D.-Q., Yang D.-W., Mai X.-T., Cheung C.Y., Chen H.-Y. (2024). Repeatability, Interocular Correlation and Agreement of Optic Nerve Head Vessel Density in Healthy Eyes: A Swept-Source Optical Coherence Tomographic Angiography Study. Int. J. Ophthalmol..

[22] Ribeiro Reis A.P., Ioannidou E., Wagner S.K., Struyven R., Sun Z., Foster P., Khawaja A.P., Petzold A., Sivaprasad S., Pontikos N. (2024). Retinal Morphology across the Menstrual Cycle: Insights from the UK Biobank. Npj Womens Health.

[23] Bayraktar M.F., Toprak G., Alkan Y. (2024). The Relationship between Choroidal Vascular Index and Non-Invasive Ultrasonographic Atherosclerosis Predictors. Photodiagnosis Photodyn. Ther..

[24] Chen L.-M., Kang M., Wang J.-Y., Xu S.-H., Chen C., Wei H., Ling Q., He L.-Q., Zou J., Wang Y.-X. (2024). Microvascular Alterations of the Ocular Surface and Retina in Connective Tissue Disease-Related Interstitial Lung Disease. Int. J. Ophthalmol..

[25] Mwale P., Zheng H., Zheng Y., Jiang B., Li Y., Wang Y., Li F., Chen X., Ke M. (2024). Detecting Pre and Early Retinal Changes in Patients with Type 2 Diabetes Mellitus Using Optical Coherence Tomography Angiography. Open J. Ophthalmol..

[26] Racioppo P., Alhasany A., Pham N.V., Wang Z., Corradetti G., Mikaelian G., Paulus Y.M., Sadda S.R., Hu Z. (2025). Automated Foveal Avascular Zone Segmentation in Optical Coherence Tomography Angiography Across Multiple Eye Diseases Using Knowledge Distillation. Bioengineering.

[27] Vagiakis I., Bakirtzis C., Andravizou A., Pirounides D. (2024). Unlocking the Potential of Vessel Density and the Foveal Avascular Zone in Optical Coherence Tomography Angiography as Biomarkers in Alzheimer’s Disease. Healthcare.

[28] Azar G., Abdelmassih Y., Bonnin S., Guindolet D., Vasseur V., Behar Cohen F., Salmon D., Mauget-Faÿsse M. (2024). Endothelial Glycocalyx Anomalies and Ocular Manifestations in Patients with Post-Acute COVID-19. J. Clin. Med..

[29] Song X., Yu Y., Zhou H., Zhang Y., Mao Y., Wang H., Cao X., Zhu X., Li Z., Li L. (2024). Acute Macular Neuroretinopathy Associated with COVID-19 Pandemic: A Real-World Observation Study. Asia-Pac. J. Ophthalmol..

[30] Bilbao-Malavé V., González-Zamora J., Saenz De Viteri M., De La Puente M., Gándara E., Casablanca-Piñera A., Boquera-Ventosa C., Zarranz-Ventura J., Landecho M.F., García-Layana A. (2021). Persistent Retinal Microvascular Impairment in COVID-19 Bilateral Pneumonia at 6-Months Follow-Up Assessed by Optical Coherence Tomography Angiography. Biomedicines.

[31] Cennamo G., Reibaldi M., Montorio D., D’Andrea L., Fallico M., Triassi M. (2021). Optical Coherence Tomography Angiography Features in Post-COVID-19 Pneumonia Patients: A Pilot Study. Am. J. Ophthalmol..

[32] Dipu T., Goel R., Arora R., Thakar M., Gautam A., Shah S., Gupta Y., Chhabra M., Kumar S., Singh K. (2022). Ocular Sequelae in Severe COVID-19 Recovered Patients of Second Wave. Indian J. Ophthalmol..

[33] El-Haddad N.S.E.-D.M., Abd El-Wahed E., Abd El-Wahab A., Shalaby S., Farag M.M.A., Mohammed N.S., Shawky S. (2023). The Effect of Post-Coronavirus Disease 2019 Infection on the Retinal Microvasculature. J. Curr. Ophthalmol..

[34] González-Zamora J., Bilbao-Malavé V., Gándara E., Casablanca-Piñera A., Boquera-Ventosa C., Landecho M.F., Zarranz-Ventura J., García-Layana A. (2021). Retinal Microvascular Impairment in COVID-19 Bilateral Pneumonia Assessed by Optical Coherence Tomography Angiography. Biomedicines.

[35] Kal M., Winiarczyk M., Cieśla E., Płatkowska-Adamska B., Walczyk A., Biskup M., Pabjan P., Głuszek S., Odrobina D., Mackiewicz J. (2022). Retinal Microvascular Changes in COVID-19 Bilateral Pneumonia Based on Optical Coherence Tomography Angiography. J. Clin. Med..

[36] Kalaw F.G.P., Warter A., Cavichini M., Knight D., Li A., Deussen D., Galang C., Heinke A., Mendoza V., Borooah S. (2023). Retinal Tissue and Microvasculature Loss in COVID-19 Infection. Sci. Rep..

[37] Koutsiaris A.G., Riri K., Boutlas S., Panagiotou T.N., Kotoula M., Daniil Z., Tsironi E.E. (2022). COVID-19 Hemodynamic and Thrombotic Effect on the Eye Microcirculation After Hospitalization: A Quantitative Case-Control Study. Clin. Hemorheol. Microcirc..

[38] Ozturk M., Kumova Guler D., Oskan E.E., Onder F. (2025). Long-Term Effects of COVID-19 on Optic Disc and Retinal Microvasculature Assessed by Optical Coherence Tomography Angiography. Diagnostics.

[39] Kazantzis D., Machairoudia G., Theodossiadis G., Theodossiadis P., Chatziralli I. (2023). Retinal Microvascular Changes in Patients Recovered from COVID-19 Compared to Healthy Controls: A Meta-Analysis. Photodiagnosis Photodyn. Ther..

[40] Saloň A., De Boever P., Goswami N. (2024). Microvascular Changes During Viral Infections: A Systematic Review of Studies Using Retinal Vessel Diameter Assessments. Biomedicines.

[41] Koutsiaris A.G., Riri K., Boutlas S., Daniil Z., Tsironi E.E. (2023). A Normative Blood Velocity Model in the Exchange Microvessels for Discriminating Health from Disease: Healthy Controls versus COVID-19 Cases. Clin. Hemorheol. Microcirc..

[42] Çakmak F., Demirbuga A., Demirkol D., Gümüş S., Torun S.H., Kayaalp G.K., Ömeroglu R.E., Somer A., Uysalol M., Yıldız R. (2021). Nailfold Capillaroscopy: A Sensitive Method for Evaluating Microvascular Involvement in Children with SARS-CoV-2 Infection. Microvasc. Res..

[43] Zharkikh E.V., Loktionova Y.I., Fedorovich A.A., Gorshkov A.Y., Dunaev A.V. (2023). Assessment of Blood Microcirculation Changes after COVID-19 Using Wearable Laser Doppler Flowmetry. Diagnostics.

[44] Karstarli Bakay O.S., Cetin N., Bakay U., Cinar G., Goksin S. (2025). A Window into the Vascular Endothelium in COVID-19: Nails. Dermatol. Pract. Concept..

[45] Sulli A., Gotelli E., Bica P.F., Schiavetti I., Pizzorni C., Aloè T., Grosso M., Barisione E., Paolino S., Smith V. (2022). Detailed Videocapillaroscopic Microvascular Changes Detectable in Adult COVID-19 Survivors. Microvasc. Res..

[46] Osiaevi I., Schulze A., Evers G., Harmening K., Vink H., Kümpers P., Mohr M., Rovas A. (2023). Persistent Capillary Rarefication in Long COVID Syndrome. Angiogenesis.

[47] Cho J.L., Villacreses R., Nagpal P., Guo J., Pezzulo A.A., Thurman A.L., Hamzeh N.Y., Blount R.J., Fortis S., Hoffman E.A. (2022). Quantitative Chest CT Assessment of Small Airways Disease in Post-Acute SARS-CoV-2 Infection. Radiology.

[48] Littlefield K.M., Watson R.O., Schneider J.M., Neff C.P., Yamada E., Zhang M., Campbell T.B., Falta M.T., Jolley S.E., Fontenot A.P. (2022). SARS-CoV-2-Specific T Cells Associate with Inflammation and Reduced Lung Function in Pulmonary Post-Acute Sequalae of SARS-CoV-2. PLoS Pathog..

[49] Dal Negro R.W., Turco P., Povero M. (2024). mRNA Vaccines Protect from the Lung Microvasculature Injury and the Capillary Blood Volume Loss Occurring in SARS-CoV-2 Paucisymptomatic Infections. Multidiscip. Respir. Med..

[50] Untersmayr E., Venter C., Smith P., Rohrhofer J., Ndwandwe C., Schwarze J., Shannon E., Sokolowska M., Sadlier C., O’Mahony L. (2024). Immune Mechanisms Underpinning Long COVID: Collegium Internationale Allergologicum Update 2024. Int. Arch. Allergy Immunol..

[51] Stein S.R., Ramelli S.C., Grazioli A., Chung J.-Y., Singh M., Yinda C.K., Winkler C.W., Sun J., Dickey J.M., Ylaya K. (2022). SARS-CoV-2 Infection and Persistence in the Human Body and Brain at Autopsy. Nature.

[52] Gáspár Z., Szabó B.G., Ceglédi A., Lakatos B. (2025). Human Herpesvirus Reactivation and Its Potential Role in the Pathogenesis of Post-Acute Sequelae of SARS-CoV-2 Infection. GeroScience.

[53] Kempuraj D., Aenlle K.K., Cohen J., Mathew A., Isler D., Pangeni R.P., Nathanson L., Theoharides T.C., Klimas N.G. (2024). COVID-19 and Long COVID: Disruption of the Neurovascular Unit, Blood-Brain Barrier, and Tight Junctions. Neuroscientist.

[54] Greene C., Connolly R., Brennan D., Laffan A., O’Keeffe E., Zaporojan L., O’Callaghan J., Thomson B., Connolly E., Argue R. (2024). Blood–Brain Barrier Disruption and Sustained Systemic Inflammation in Individuals with Long COVID-Associated Cognitive Impairment. Nat. Neurosci..

[55] Qiao H., Deng X., Qiu L., Qu Y., Chiu Y., Chen F., Xia S., Muenzel C., Ge T., Zhang Z. (2024). SARS-CoV-2 Induces Blood-brain Barrier and Choroid Plexus Barrier Impairments and Vascular Inflammation in Mice. J. Med. Virol..

[56] Reiken S., Sittenfeld L., Dridi H., Liu Y., Liu X., Marks A.R. (2022). Alzheimer’s-like Signaling in Brains of COVID-19 Patients. Alzheimers Dement..

[57] Charnley M., Islam S., Bindra G.K., Engwirda J., Ratcliffe J., Zhou J., Mezzenga R., Hulett M.D., Han K., Berryman J.T. (2022). Neurotoxic Amyloidogenic Peptides in the Proteome of SARS-CoV2: Potential Implications for Neurological Symptoms in COVID-19. Nat. Commun..

[58] Peluso M.J., Deeks S.G., Mustapic M., Kapogiannis D., Henrich T.J., Lu S., Goldberg S.A., Hoh R., Chen J.Y., Martinez E.O. (2022). SARS-CoV-2 and Mitochondrial Proteins in Neural-Derived Exosomes of COVID-19. Ann. Neurol..

[59] Díaz-Resendiz K.J.G., Benitez-Trinidad A.B., Covantes-Rosales C.E., Toledo-Ibarra G.A., Ortiz-Lazareno P.C., Girón-Pérez D.A., Bueno-Durán A.Y., Pérez-Díaz D.A., Barcelos-García R.G., Girón-Pérez M.I. (2022). Loss of Mitochondrial Membrane Potential (Δ *Ψ* m) in Leucocytes as Post-COVID-19 Sequelae. J. Leukoc. Biol..

[60] Zhang B.-Z., Chu H., Han S., Shuai H., Deng J., Hu Y., Gong H., Lee A.C.-Y., Zou Z., Yau T. (2020). SARS-CoV-2 Infects Human Neural Progenitor Cells and Brain Organoids. Cell Res..

[61] Oaklander A.L., Mills A.J., Kelley M., Toran L.S., Smith B., Dalakas M.C., Nath A. (2022). Peripheral Neuropathy Evaluations of Patients With Prolonged Long COVID. Neurol. Neuroimmunol. Neuroinflamm..

[62] Larsen N.W., Stiles L.E., Shaik R., Schneider L., Muppidi S., Tsui C.T., Geng L.N., Bonilla H., Miglis M.G. (2022). Characterization of Autonomic Symptom Burden in Long COVID: A Global Survey of 2,314 Adults. Front. Neurol..

[63] Campen C.L.M.C.V., Visser F.C. (2022). Long-Haul COVID Patients: Prevalence of POTS Are Reduced but Cerebral Blood Flow Abnormalities Remain Abnormal with Longer Disease Duration. Healthcare.

[64] Tavee J. (2024). Current Concepts in Long COVID-19 Brain Fog and Postural Orthostatic Tachycardia Syndrome. Ann. Allergy. Asthma. Immunol..

[65] Fedorowski A., Fanciulli A., Raj S.R., Sheldon R., Shibao C.A., Sutton R. (2024). Cardiovascular Autonomic Dysfunction in Post-COVID-19 Syndrome: A Major Health-Care Burden. Nat. Rev. Cardiol..

[66] Yeoh Y.K., Zuo T., Lui G.C.-Y., Zhang F., Liu Q., Li A.Y., Chung A.C., Cheung C.P., Tso E.Y., Fung K.S. (2021). Gut Microbiota Composition Reflects Disease Severity and Dysfunctional Immune Responses in Patients with COVID-19. Gut.

[67] Liu Q., Mak J.W.Y., Su Q., Yeoh Y.K., Lui G.C.-Y., Ng S.S.S., Zhang F., Li A.Y.L., Lu W., Hui D.S.-C. (2022). Gut Microbiota Dynamics in a Prospective Cohort of Patients with Post-Acute COVID-19 Syndrome. Gut.

[68] König R.S., Albrich W.C., Kahlert C.R., Bahr L.S., Löber U., Vernazza P., Scheibenbogen C., Forslund S.K. (2022). The Gut Microbiome in Myalgic Encephalomyelitis (ME)/Chronic Fatigue Syndrome (CFS). Front. Immunol..

[69] Pretorius E., Venter C., Laubscher G.J., Kotze M.J., Oladejo S.O., Watson L.R., Rajaratnam K., Watson B.W., Kell D.B. (2022). Prevalence of Symptoms, Comorbidities, Fibrin Amyloid Microclots and Platelet Pathology in Individuals with Long COVID/Post-Acute Sequelae of COVID-19 (PASC). Cardiovasc. Diabetol..

[70] Bellone S., Siegel E.R., Scheim D.E., Santin A.D. (2024). Increased von Willebrand and Factor VIII Plasma Levels in Gynecologic Patients with Post-Acute-COVID-Sequela (PASC)/Long COVID. Gynecol. Oncol. Rep..

[71] Popazu C., Romila A., Petrea M., Grosu R.M., Lescai A.-M., Vlad A.L., Oprea V.D., Baltă A.A.Ș. (2025). Overview of Inflammatory and Coagulation Markers in Elderly Patients with COVID-19: Retrospective Analysis of Laboratory Results. Life.

[72] Baldassarro V.A., Alastra G., Cescatti M., Quadalti C., Lorenzini L., Giardino L., Calzà L. (2024). SARS-CoV-2-Related Peptides Induce Endothelial-to-Mesenchymal Transition in Endothelial Capillary Cells Derived from Different Body Districts: Focus on Membrane (M) Protein. Cell Tissue Res..

[73] Gultom M., Lin L., Brandt C.B., Milusev A., Despont A., Shaw J., Döring Y., Luo Y., Rieben R. (2024). Sustained Vascular Inflammatory Effects of SARS-CoV-2 Spike Protein on Human Endothelial Cells. Inflammation.

[74] Šuligoj T., Coombes N.S., Booth C., Savva G.M., Bewley K.R., Funnell S.G.P., Juge N. (2024). Modelling SARS-CoV-2 Infection in a Human Alveolus Microphysiological System. Access Microbiol..

[75] Romanowska-Kocejko M., Braczko A., Jędrzejewska A., Żarczyńska-Buchowiecka M., Kocejko T., Kutryb-Zając B., Hellmann M. (2025). Follow-up Assessment of the Microvascular Function in Patients with Long COVID. Microvasc. Res..

[76] Valencia I., Lumpuy-Castillo J., Magalhaes G., Sánchez-Ferrer C.F., Lorenzo Ó., Peiró C. (2024). Mechanisms of Endothelial Activation, Hypercoagulation and Thrombosis in COVID-19: A Link with Diabetes Mellitus. Cardiovasc. Diabetol..

[77] Ståhlberg M., Fischer K., Tahhan M., Zhao A., Fedorowski A., Runold M., Nygren-Bonnier M., Björnson M., Lund L.H., Bruchfeld J. (2024). Post-Acute COVID-19 Syndrome: Prevalence of Peripheral Microvascular Endothelial Dysfunction and Associations with NT-ProBNP Dynamics. Am. J. Med..

[78] Aird W.C. (2007). Phenotypic Heterogeneity of the Endothelium: I. Structure, Function, and Mechanisms. Circ. Res..

[79] Koutsiaris A.G. (2016). Wall Shear Stress in the Human Eye Microcirculation in Vivo, Segmental Heterogeneity and Performance of in Vitro Cerebrovascular Models. Clin. Hemorheol. Microcirc..

[80] Koutsiaris A.G. (2009). A Velocity Profile Equation for Blood Flow in Small Arterioles and Venules of Small Mammals in Vivo and an Evaluation Based on Literature Data. Clin. Hemorheol. Microcirc..

[81] Mierke C.T. (2024). Mechanosensory Entities and Functionality of Endothelial Cells. Front. Cell Dev. Biol..

[82] Krüger-Genge A., Blocki A., Franke R.-P., Jung F. (2019). Vascular Endothelial Cell Biology: An Update. Int. J. Mol. Sci..

[83] Boegehold M.A. (1998). Heterogeneity of Endothelial Function within the Circulation. Curr. Opin. Nephrol. Hypertens..

[84] Cutolo M., Sulli A., Smith V., Gotelli E. (2023). Emerging Nailfold Capillaroscopic Patterns in COVID-19: From Acute Patients to Survivors. Reumatismo.

[85] Koutsiaris A.G. (2024). A Blood Supply Pathophysiological Microcirculatory Mechanism for Long COVID. Life.

[86] Koutsiaris A.G. (2023). The Velocity-Diffusion Equation in the Exchange Microvessels. Clin. Hemorheol. Microcirc..

[87] Liu P., Ernst T., Liang H., Jiang D., Cunningham E., Ryan M., Lu H., Kottilil S., Chang L. (2024). Elevated Cerebral Oxygen Extraction in Patients with Post-COVID Conditions. NeuroImmune Pharmacol. Ther..

[88] Romanowska-Kocejko M., Jędrzejewska A., Braczko A., Stawarska K., Król O., Frańczak M., Harasim G., Smoleński R.T., Hellmann M., Kutryb-Zając B. (2024). Red Blood Cell Adenylate Energetics Is Related to Endothelial and Microvascular Function in Long COVID. Biomedicines.

[89] Jamieson A., Al Saikhan L., Alghamdi L., Hamill Howes L., Purcell H., Hillman T., Heightman M., Treibel T., Orini M., Bell R. (2024). Mechanisms Underlying Exercise Intolerance in Long COVID: An Accumulation of Multisystem Dysfunction. Physiol. Rep..

[90] Lafetá M.L., Souza V.C., Menezes T.C.F., Verrastro C.G.Y., Mancuso F.J., Albuquerque A.L.P., Tanni S.E., Izbicki M., Carlstron J.P., Nery L.E. (2023). Exercise Intolerance in Post-Coronavirus Disease 2019 Survivors after Hospitalisation. ERJ Open Res..

[91] Russell S.L., Okwose N.C., Rahman M., Lee B.J., McGregor G., Raleigh S.M., Sandhu H., Roden L.C., Banerjee P., Jakovljevic D.G. (2025). The Effect of COVID-19 on Cardiovascular Function and Exercise Tolerance in Healthy Middle-Age and Older Individuals. Scand. Cardiovasc. J..

[92] Kell D.B., Pretorius E. (2022). The Potential Role of Ischaemia–Reperfusion Injury in Chronic, Relapsing Diseases Such as Rheumatoid Arthritis, Long COVID, and ME/CFS: Evidence, Mechanisms, and Therapeutic Implications. Biochem. J..

[93] Wirth K.J., Löhn M. (2024). Microvascular Capillary and Precapillary Cardiovascular Disturbances Strongly Interact to Severely Affect Tissue Perfusion and Mitochondrial Function in Myalgic Encephalomyelitis/Chronic Fatigue Syndrome Evolving from the Post COVID-19 Syndrome. Medicina.

[94] Panagiotides N.G., Poledniczek M., Andreas M., Hülsmann M., Kocher A.A., Kopp C.W., Piechota-Polanczyk A., Weidenhammer A., Pavo N., Wadowski P.P. (2024). Myocardial Oedema as a Consequence of Viral Infection and Persistence—A Narrative Review with Focus on COVID-19 and Post COVID Sequelae. Viruses.

[95] Theresa C., Katebe B., Shibao C.A., Kirabo A. (2024). Arterial Stiffness in Adults with Long COVID in sub-Saharan Africa. Physiol. Rep..

[96] Goldstein R.E., Hulten E.A., Arnold T.B., Thomas V.M., Heroy A., Walker E.N., Fox K., Lee H., Libbus J., Markos B. (2024). Exercise Stress Echocardiography Shows Impaired Left Ventricular Function after Hospitalization with COVID-19 without Overt Myocarditis: A Pilot Study. Physiol. Rep..

[97] Passos C.R., Moreira A.A., Reis R.F., Dos Santos R.W., Lobosco M., Rocha B.M. (2025). A Coupled Model of the Cardiovascular and Immune Systems to Analyze the Effects of COVID-19 Infection. BioTech.

